# Evaluation of the proliferation marker Ki-67 in gliomas: Interobserver variability and digital quantification

**DOI:** 10.1186/s13000-018-0711-2

**Published:** 2018-06-09

**Authors:** Ljudmilla A. G. Nielsen, Julie A. Bangsø, Kim H. Lindahl, Rikke H. Dahlrot, Jacob v. B. Hjelmborg, Steinbjørn Hansen, Bjarne W. Kristensen

**Affiliations:** 1 Department of Pathology, Odense University Hospital, J. B. Winsløws Vej 15, Entrance 240, DK-5000 Odense C, Denmark; 2 Department of Pathology, Hospital of Southern Jutland/ Sygehus Sønderjylland, Kresten Philipsens Vej 15, Dk-6200 Aabenraa, Denmark; 3 Department of Oncology, Odense University Hospital, Sdr. Boulevard 29, Dk-5000 Odense C, Denmark; 4 Department of Public Health, Epidemiology, Biostatistics and Biodemography, University of Southern Denmark, J.B. Winsløws Vej 9, Entrance B, 1st, Dk-5000 Odense C, Denmark; 5 Department of Clinical Research, University of Southern Denmark, J. B. Winsløws Vej 15, Entrance 240, DK-5000 Odense C, Denmark

**Keywords:** Glioblastoma, Astrocytoma, Digital analysis, Whole slides, Labelling index

## Abstract

**Background:**

The Ki-67 Labelling Index (LI) is used as an ancillary tool in glioma diagnostics. Interobserver variability has been reported and no precise guidelines are available. Nor is it known whether novel digital approaches would be an advantage. Our aim was to evaluate the inter- and intraobserver variability of the Ki-67 LI between two pathologists and between pathologists and digital quantification both in whole tumour slides and in hot spots using narrow but diagnostically relevant intervals.

**Methods:**

In samples of 235 low and high grade gliomas, two pathologists (A and B) estimated the Ki-67 LI (5–10% intervals) for whole tumour slides and for hot spots. In 20 of the cases intraobserver variability was evaluated. For digital quantification (C) slides were scanned with subsequent systematic random sampling of viable tumour areas. A software classifier trained to identify positive and negative nuclei calculated the Ki-67 LI. The interobserver agreements were evaluated using kappa (κ) statistics.

**Results:**

The observed proportions of agreement and κ values for Ki-67 LI for whole tumour slides were: A/B: 46% (κ = 0.32); A/C: 37% (κ = 0.26); B/C: 37% (κ = 0.26). For hot spots equivalent values were: A/B: 14% (κ = 0.04); A/C: 18% (κ = 0.09); B/C: 31% (κ = 0.21).

**Conclusions:**

Interobserver variability was pronounced between pathologists and for pathologists versus digital quantification when attempting to estimate a precise value of the Ki-67 LI. Ki-67 LI should therefore be used with caution and should not be over interpreted in the grading of gliomas. Digital quantification of Ki-67 LI in gliomas was feasible, but intra- and interlaboratory robustness need to be determined.

**Electronic supplementary material:**

The online version of this article (10.1186/s13000-018-0711-2) contains supplementary material, which is available to authorized users.

## Background

Gliomas are the most frequent primary brain tumours, of which glioblastomas are the most malignant [1]. According to the WHO guidelines, gliomas are graded I-IV using the following histological criteria: cytological atypia, mitotic activity, cellularity, microvascular proliferation and/or necrosis [1]. Mitotic activity is included in the distinction between grade II and grade III gliomas, whereas microvascular proliferation and/or necrosis suggest high grade gliomas like anaplastic oligodendroglioma (grade III) or glioblastoma (grade IV) [1]. To limit inter- and intraobserver variability in the histological grading of gliomas [2] ancillary diagnostic tools are needed, especially in small tumour samples [3].

The proliferation marker Ki-67 (or MIB-1) has been suggested as an ancillary marker [1, 3] in the grading of gliomas. The Ki-67 protein is located in the cell nucleus and can be detected in the active phases of the cell cycle, whereas it cannot be detected in the quiescent phase G0 [4]. The so-called Ki-67 labelling index (LI) being defined as the percentage of Ki-67 positive tumour nuclei of all tumour nuclei correlates with the histological tumour grade [5, 6]. The general Ki-67 LI values reported by the WHO for gliomas are below 4% for diffuse astrocytomas, and between 5 and 10% for anaplastic astrocytomas, while the reported mean values for glioblastomas are between 15 and 20% [1]. There is, however, an overlap of Ki-67 LIs between histological grades [5, 7], and the reported Ki-67 LI values vary between studies [5]. Part of this overlap may be explained by the approaches used for estimating the Ki-67 LI, as no precise guidelines exist and considerable interobserver variability has been reported [8]. Therefore a more standardized approach for the evaluation of the Ki-67 LI is warranted [9].

Several methods, including digital approaches, have been proposed to evaluate the Ki-67 LI in gliomas and other neoplasms of the central nervous system [3, 6, 10–14]. In gliomas, a common approach is to focus on the tumour area with the most intense proliferation – the so-called hot spot - and to estimate by various manual methods the Ki-67 LI [3, 7, 8, 10, 15]. Limited attention has been paid to the value of whole tumour slide mean values. As gliomas may be heterogeneous the area with most intense proliferation may not be readily identifiable. Neither is it certain that the most intense hot spot is represented in the biopsy or in the section chosen for immunohistochemical staining. With conventional evaluation it may be impossible to assess a whole tumour slide unless one uses a semi quantitative approach. With novel digital advances it might be possible to assess larger tumour areas, with possibly better interobserver agreement.

The aim of this study was to evaluate the inter- and intraobserver variability of the Ki-67 LI between two pathologists and between pathologists and a digital quantitative approach. The pathologists used a semi-quantitative approach. Both whole tumour slide mean values and hot spot values were evaluated for both approaches.

A poster with preliminary results has been presented [16].

## Methods

### Patient population

The study was based on a cohort of adult patients with primary glioma with no previous treatment diagnosed between 2005 and 2009. The cohort has been used in various biomarker studies [17–22]. Formalin-fixed paraffin-embedded tissue blocks from the primary surgery were used and sufficient tissue was available for immunohistochemical staining in 235 cases. The gliomas included grade I (3 cases), grade II (25 cases), grade III (25 cases) and grade IV tumours (182 cases). The histological diagnoses of the cases were pilocytic astrocytoma (grade I), diffuse astrocytoma, oligodendroglioma, oligoastrocytoma (grade II), anaplastic astrocytoma, anaplastic oligodendroglioma, anaplastic oligoastrocytoma (grade III), glioblastoma and gliosarcoma (grade IV).

### Immunohistochemical procedure

For immunohistochemical staining a 3 μm section was cut from each tissue block. All stained sections were whole tumour sections. The immunostaining was performed on the automated immunohistochemical staining system BenchMark Ultra (Ventana medical system, Inc., USA), and all sections were stained in the same run or in runs following one another. All reagents used were from Ventana Medical System, Inc., USA. Antigen retrieval was achieved using heat induced epitope retrieval for 48 min with a maximum temperature of 100 °C and Cell Conditioner 1 as the buffer. The primary antibody used was monoclonal rabbit anti-Ki-67 (30–9), ready to use dilution, with an incubation time of 12 min at 36 °C. The visualization system was OptiView DAB and counterstaining with Hematoxylin II and Blue Reagent followed immunostaining. Nuclear staining was considered positive. Appropriate staining of the germinal centres and squamous epithelium of normal tonsil served as a positive control, with superficial epithelial cells representing a negative tissue control. Moreover, omission of the anti-Ki-67 antibody served as a negative control.

### Conventional microscopic evaluation

Two pathologists made a visual estimate of the Ki-67 LI using a light microscope, resembling daily practice. No fixed number of nuclei was counted. This approach was used in order to reflect daily diagnostics. The Ki-67 LI was defined as the percentage of positive tumour nuclei of all tumour nuclei. Necrosis, normal brain tissue, zones of infiltration, endothelial and inflammatory cells were omitted from the evaluation. A whole slide mean value and a hot spot value were estimated for each tumour in independent sessions.

To estimate the whole slide mean values, the tumour slides were viewed using the scanning objective (40× magnification) with subsequently evaluation at higher magnification of selected foci of viable tumour areas (100× magnification). The number of selected foci was not defined beforehand in order to reflect daily diagnostics. Each pathologist chose how many microscopic fields to examine in each case. This number varied between cases depending on the area of the tumour tissue on the slide and the heterogeneity of the tumour tissue.

A hot spot was defined as the area with the most intense proliferation covering an area of at least one high power field (HPF = 0.24 mm^2^) at 400× magnification. Selection of hot spots was done by scanning the slide at low magnification, and the hot spot was selected in the area with the highest LI. In each case one hot spot was chosen for evaluation.

The visual estimates of the Ki-67 LIs were recorded using the following pre-defined categories: 0, 5, 10, 15, 20, 25, 30, 40, 50, 60, 70, 80, 90 and 100%.

Twenty slides were re-evaluated to assess the intraobserver variability.

### Digital quantification

For the digital quantification whole slides were scanned using a slide scanner with a 20× objective (Hamamatsu NanoZoomer 2.0-HT, Ballerup, Denmark). The Visiopharm integrator system software (Visiopharm, Hørsholm, Denmark) was used for the digital image analysis. Tumour tissue was outlined manually and necrosis, vessels, invasion zones and normal brain tissue were excluded. Images to be quantified were obtained by systematic random sampling of 5% of the tissue [e.g. if the area of tissue on a slide was Amm^2^, then 0.05 x Amm^2^ would be selected randomly from the slide]. The area of tissue on the slide differed between cases, leading therefore to different numbers of sample images between the cases. A minimum of 5 sample images per slide, each with at least 50% of viable tumour tissue was accepted as sufficient for further analysis. In cases with only sparse tumour tissue available, the slide was resampled using a sample fraction of 20% and a minimum of three sample images was accepted [e.g. if 0.05 x Amm^2^ resulted in only 5 sample images with less than 50% of viable tumour tissue on each sample image, or if 0.05 x Amm^2^ resulted in less than 5 sample images, the whole slide would be resampled. An area of 0.20 x Amm^2^ would then be selected randomly from the slide]. Using the Tissuemorph software, a classifier was trained to detect Ki-67 positive and negative nuclei. This was done by two of the authors including a neuropathologist on the basis of morphology, variation of staining colour of the nuclei and background staining. The Ki-67 LI was then calculated automatically. The mean value included all sample images of a tumour slide. A hot spot was defined as the sample image with the highest Ki-67 LI.

The procedure was repeated for 20 slides to assess the intraobserver variability.

### Statistics

The continuous data of the digital quantification were grouped in intervals corresponding to the pre-defined categories used for the conventional evaluation. The values of the Ki-67 LI were assessed pairwise for: Pathologist A vs. Pathologist B, Digital quantification vs. Pathologist A, Digital quantification vs. Pathologist B. The interobserver variability was evaluated using Cohen’s kappa (κ) for two raters. The kappa statistic is calculated by comparing the observed proportion of agreement with the agreement that would be expected by chance [23]. The statistical programme used was R version 3.0.2 [24] with Package ‘irr’ [25], including R Studio version 0.98.1091 [26].

### Ethics

The project was approved by the Local Committee on Health Research Ethics and the Danish Data Protection Agency. According to the Danish Tissue Application Register, use of the tissue was not prohibited.

## Results

The pairs of observations distributed on the pre-defined categories and κ values are shown in Tables 1, 2, 3 and 4. The diagonal represents agreement (a difference between two observations of zero categories). One shift to the side horizontally or vertically from the diagonal indicates a difference of one category between two observations. Two shifts from the diagonal indicate a difference of two categories etc.

**Table 1:** Whole tumour slide mean values of Ki-67 LI for two pathologists

Pathologist A												
	%	0	5	10	15	20	25	30	40	50	60	Total (n)
Pathologist B	0	**14**	7									21
	5	3	**21**	21	8	1						54
	10			**29**	23	5	2	1				60
	15		1	8	**33**	9	3					54
	20				16	**9**	2	1				28
	25				2	5		1	1			9
	30				1	3	2			1		7
	40								**1**			1
	50											
	60									1		1
	Total (n)	17	29	58	83	32	9	3	2	2		235
κ												0.32
SE κ												0.03

*n*: Number of cases

*SE*: Standard error

Cases marked in **bold** indicate agreement

**Table 2:** Whole tumour slide mean values of Ki-67 LI for two raters

Digital quantification												
	%	0	5	10	15	20	25	30	40	50	60	Total (n)
Pathologist A	0	**11**	5	1								17
	5	10	**13**	5	1							29
	10		8	**30**	13	6	1					58
	15		3	9	**23**	19	22	6	1			83
	20		1		3	**6**	10	9	3			32
	25					1	**2**	2	4			9
	30					1			2			3
	40								**2**			2
	50								1	**1**		2
	60											
	Total (n)	21	30	45	40	33	35	17	13	1		235
κ												0.26
SE κ												0.03
Pathologist B	0	**15**	5	1								21
	5	6	**22**	24	1	1						54
	10		3	**18**	19	14	3	3				60
	15			2	**19**	12	16	4	1			54
	20				1	**6**	13	5	3			28
	25						**3**	2	4			9
	30							**3**	4			7
	40								**1**			1
	50											
	60									1		1
	Total (n)	21	30	45	40	33	35	17	13	1		235
κ	0.26											
SE κ	0.03											

*n* Number of cases

*SE* Standard error

Cases marked in **bold** indicate agreement

**Table 3:** Hot spot values of Ki-67 LI for two pathologists

Pathologist A															
	%	0	5	10	15	20	25	30	40	50	60	70	80	90	Total (n)
Pathologist B	0	**5**	4	3											12
	5		**3**	15	5										23
	10		1	**2**	14	7	1	2	1						28
	15				**2**	8	12	10	6	1					39
	20				2	**4**	2	20	15	4					47
	25				1		**6**	7	9	5					28
	30							**3**	15	12	4				34
	40						1	3	**3**	4	1	1			13
	50								1	**4**	2	1			8
	60														
	70														
	80											2			2
	90												1		1
	Total (n)	5	8	20	24	19	22	45	50	30	7	4	1		235
κ															0.04
SE κ															0.02

*n* Number of cases

*SE* Standard error

Cases marked in **bold** indicate agreement

**Table 4:** Hot spot values of Ki-67 LI for two raters

Digital quantification															
	%	0	5	10	15	20	25	30	40	50	60	70	80	90	Total (n)
Pathologist A	0	**4**					1								5
	5	1	**2**	4			1								8
	10		9	**6**	1	1	1		1			1			20
	15		2	3	**5**	6	1	3	4						24
	20				6	**5**	2	4	2						19
	25	1		1	1	4	**4**	5	6						22
	30		2	2	2	5	7	**14**	10	2	1				45
	40				2	4	4	10	**19**	6	5				50
	50		1			1	1	3	12	**8**	2	2			30
	60								1	1	**3**	1	1		7
	70								1		1	**2**			4
	80											1			1
	90														
	Total (n)	6	16	16	17	26	22	39	56	17	12	7	1		235
κ															0.21
SE κ															0.02
Pathologist B	0	**4**	1	3		1	2		1						12
	5	1	**12**	6	2	1			1						23
	10			**5**	7	7	4	2	2			1			28
	15			1	**6**	10	6	5	11						39
	20		1	1	1	**2**	5	23	9	3	2				47
	25	1	2			3	**3**	6	8	2	3				28
	30				1	1		**2**	18	7	4	1			34
	40						2	1	**5**	2	2	1			13
	50					1				**3**	1	2	1		8
	60														
	70														
	80								1			1			2
	90											1			1
	Total (n)	6	16	16	17	26	22	39	56	17	12	7	1		235
κ															0.09
SE κ															0.02

*n* Number of cases

*SE* Standard error

Cases marked in **bold** indicate agreement

### Interobserver variability

#### Whole tumour slide mean values

**Fig. 1:**
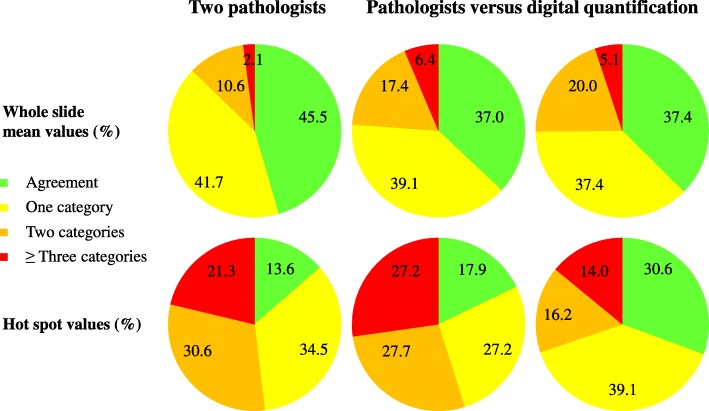
Comparison of interobserver variation. The pairwise observed proportions of agreement are shown as green areas. The interobserver variation is distributed on the proportion of observations with a difference of one category (yellow areas), two categories (orange areas) and three categories or more (red areas) respectively

The median [range] Ki-67 LI for the two pathologists (A and B) and for the digital approach (C) were: A: 10% [0–60%]; B: 15% [0–50%]; C: 14% [0–51%] (Tables 1 and 2). The pairwise proportions of agreement varied from 37 to 46% (Fig. 1, green areas), while the corresponding κ values were in the range 0.26–0.32 (Tables 1 and 2). In 52–57% of cases there was an interobserver variation of the estimated Ki-67 LI of one or two categories (Fig. 1, yellow and orange areas), while there was a pairwise difference in estimated values of three categories or more in 2–6% of cases (Fig. 1, red areas). The largest difference in estimated values both between two pathologists and between a pathologist and digital quantification were four categories (Tables 1 and 2): e.g. in one case Pathologist A estimated the Ki-67 LI to be 30%, while Pathologist B estimated the Ki-67 LI to be 10% in the same case. In three cases the Ki-67 LI was 30% as a result of the digital quantification, while Pathologist B evaluated the Ki-67 LI to be 10% in the same three cases.

#### Hot spot values

The median [range] Ki-67 LI for pathologists (A and B) and for the digital approach (C) were: A: 20% [0–90%]; B: 30% [0–80%]; C: 31% [0–81%] (Tables 3 and 4). The pairwise proportions of agreement varied from 14 to 31% (Fig. 1, green areas), while the corresponding κ values were in the range 0.04–0.21 (Tables 3 and 4). In 55–65% of cases there was an interobserver variation of the estimated Ki-67 LI of one or two categories (Fig. 1, yellow and orange areas), while there was a pairwise difference in estimated values of three categories or more in 14–27% of cases (Fig. 1). The largest difference in estimated values between two pathologists was five categories, and the largest difference in estimated values between a pathologist and digital quantification was eight categories (Tables 3 and 4): e.g. in one case Pathologist A evaluated the Ki-67 LI to be 40%, while Pathologist B evaluated the Ki-67 LI to be 10% in the same case. In one case the Ki-67 LI was 70% for the digital quantification, while Pathologist A estimated the Ki-67 LI to be 10% in the same case.

### Intraobserver variability

#### Whole tumour slide mean values

For the two pathologists there was agreement for A in 13/20 cases (Additional file 1: Table S1) and for B in 8/20 cases (Additional file 1: Table S2). There was a variation of one category between the two estimates of the Ki-67 LI for A in 6/20 cases and for B in 11/20 cases. A difference of two categories was found in 1/20 cases for each of the pathologists. For the digital quantifications there was less than 5% difference in 18/20 cases (Additional file 1: Table S5). In one case there was 8.6% difference and in one case there was 12.2% difference between the two quantifications.

#### Hot spot values

For the pathologists there was agreement for A in 9/20 cases (Additional file 1: Table S3) and for B in 7/20 cases (Additional file 1: Table S4). A difference of one category was found for A in 9/20 cases and for B in 5/20 cases. Difference of two categories was found for B in 4/20 cases. Difference of three categories or more was found for A in 2/20 cases and for B in 4/20 cases. For the digital quantifications there was less than 5% difference in 12/20 cases (Additional file 1: Table S5). There was more than 5% and less than 10% difference in 4/20 cases. In the last four cases the difference was 11.3, 20.8, 21.2 and 34.6% respectively.

## Discussion

We found that an attempt of precise estimation of the Ki-67 LI in gliomas using narrow but diagnostically relevant intervals resulted in pronounced interobserver variability. The highest kappa value was 0.32 and the lowest kappa value had a level corresponding to what could be expected by chance. Some cases accordingly produced a large difference between estimated values. The digital approach showed a tendency for higher Ki-67 LI values when evaluating whole tumour slides. In terms of interobserver variability we found no difference between two pathologists and the pathologists compared to the digital approach.

A high level of interobserver variability for the Ki-67/ MIB1 LI between pathologists has previously been demonstrated in gliomas [8]. Some authors report that the interobserver variability in gliomas may be reduced when using specific methods of counting [10]. In a study of oligodendrogliomas the authors report the overall agreement to be good, with a few cases resulting in marked interobserver variability [27]. For intracranial ependymomas different levels of interobserver variability has been reported, and the kappa values were found to depend on the cut off level chosen and on the level of experience of the observers [28]. In the present study we had chosen to use narrow intervals for the estimated Ki-67 LI being however comparable to the levels suggested to guide the grading of gliomas. When using the kappa statistics, many categories will tend to result in lower kappa values [23]. In several studies the results were dichotomized using specific cut-off values [8, 10, 28] explaining that kappa values in general were found to be higher than in our study. A limitation of our study is that the results are based on the observations of only two pathologists. Nevertheless it reflects the daily situation in many units of neuropathology.

In our study, the interobserver variability of the Ki-67 LI was highest, when evaluating hot spots, indicating that in terms of interobserver variability, hot spot values, especially, should be used with caution. The literature is scarce with regard to evaluation of the Ki-67 LI in gliomas in whole tumour slides. One study of gliomas, however, found that a semi quantitative evaluation of the MIB-1 LI, where whole tumour slides were rated as low, intermediate and high, resulted in a general agreement among observers [8].

In our assessment of intraobserver variability the best results were achieved for the digital method, when evaluating the mean values of whole tumour slides. All except two cases showed a difference of less than 5% between the two evaluations being the equivalent of an intraobserver variation of zero categories (agreement) for pathologists. Because of the small number of cases no statistical analysis was done. The sampling of the tissue on the slide was performed by systematic random sampling and thus different sample areas could be quantified when repeating the evaluation. This can explain why the difference of the Ki-67 LI exceeded 5% in two cases. A preliminary test considering reproducibility and time consumption resulted in the choice of a sample fraction of 5% in the present study. In the preliminary test there was no remarkable improvement in terms of reproducibility when using a larger sample fraction except in cases with only little amount of tissue on the slide. Using a larger sample fraction of the digitalised slide or quantifying all the viable tumour areas would be very time consuming because of the large number of sample images per slide combined with the manual step of outlining tumour tissue. In previous studies various digital techniques have been found to be feasible in the determination of the Ki-67 LI in gliomas and meningiomas [11, 13, 29], including detection of hot spot areas [30, 31]. In our study, the intraobserver variability for the digital quantification of hot spot values resulted in 8 cases with a difference larger than 5% between Ki-67 LI values. The digital quantification method we used was performed by systematic random sampling of the tissue and was thus not designed specifically to identify the areas with the highest Ki-67 LI. These areas would not necessarily be included in the sample images. The hot spot values for the digital quantification was a result of a single sample image making this method very sensitive to the random area represented in that sample image. This might explain the larger variability for hot spot values compared to whole slide mean values. This might also explain some of the conspicuous differences in evaluated hot spot values when comparing the digital quantification with the pathologists. We find that the digital quantification method provides a possibility of assessing larger tissue areas with numerous cells with possibly more accuracy as compared to the conventional method. Challenges using digital quantification based on a classifier include the presence of both false positive and false negative nuclei [11]. We found that false positive nuclei could be a result of labelling of non-tumour nuclei in inflammatory cells or vessels. False negative nuclei could result from weak staining or from merging of tumour cell nuclei because of physical overlap. These challenges, however, will also be met by the pathologist. The digital method requires additional steps including the scanning of slides and the outlining of relevant tumour areas prior to the evaluation process, whereas the conventional method is directly applicable in the daily diagnostic practice. In a fully digitalised diagnostic setting the digital quantification would be easily implemented. The high proportion of intraobserver agreement for the digital quantification in case of whole tumour slide mean values supports that the classifier used in this study is fairly accurate. In our study only twenty cases were re-evaluated and in order to test the accuracy of the classifier, larger studies of the reproducibility are needed. If our approach were used in other laboratories, it would be important to test the reproducibility within each laboratory, but also to determine the reproducibility between laboratories. It should be emphasized that the Ki-67 staining procedure itself should also be standardized to reach a high intra- and interlaboratory reproducibility.

## Conclusions

In conclusion our results suggest marked interobserver variability when attempting to estimate precise values for the Ki-67 LI. We find that such precise values should be used with caution and such values should therefore not be over interpreted in the grading of gliomas. The digital quantification was feasible, but intra- and interlaboratory robustness of the method need to be determined.

## Additional file

Additional file 1: The results of the intraobserver variability. **Table S1** Intraobserver variability for pathologist A - Whole tumour slide mean values of Ki-67 LI. **Table S2** Intraobserver variability for pathologist B - Whole tumour slide mean values of Ki-67 LI. **Table S3** Intraobserver variability for pathologist A – Hot spot values of Ki-67 LI. **Table S4** Intraobserver variability for pathologist B – Hot spot values of Ki-67 LI. **Table S5** Intraobserver variability for the digital quantification of the Ki-67 LI (%). (DOCX 27 kb)

## Abbreviations

HPF: High power field
LI: Labelling Index
SE: Standard error
κ: Kappa
