# Supplementary material for: Evaluation of the proliferation marker Ki-67 in gliomas: Interobserver variability and digital quantification
Source: Diagn Pathol. 2018 Jun 9;13:38. doi: 10.1186/s13000-018-0711-2 (PMC5994254; doi:10.1186/s13000-018-0711-2)
Supplement: Supplementary file 1 — The results of the intraobserver variability. Table S1 Intraobserver variability for pathologist A - Whole tumour slide mean values of Ki-67 LI. Table S2 Intraobserver variability for pathologist B - Whole tumour slide mean values of Ki-67 LI. Table S3 Intraobserver variability for pathologist A – Hot spot values of Ki-67 LI. Table S4 Intraobserver variability for pathologist B – Hot spot values of Ki-67 LI. Table S5 Intraobserver variability for the digital quantification of the Ki-67 LI (%). (DOCX 27 kb) [file 13000_2018_711_MOESM1_ESM.docx]

**Supplementary tables: Intraobserver variability**

| **Table S1** Intraobserver variability for pathologist A - Whole tumour slide mean values of Ki-67 LI | | | | | | | | | |
| --- | --- | --- | --- | --- | --- | --- | --- | --- | --- |
| % | 0 | 5 | 10 | 15 | 20 | 25 | 30 | 40 | Total (n) |
| 0 | **2** | 1 |  |  |  |  |  |  | 3 |
| 5 |  | **3** |  |  |  |  |  |  | 3 |
| 10 |  |  | **2** |  |  |  |  |  | 2 |
| 15 |  |  | 1 | **5** | 1 |  |  |  | 7 |
| 20 |  |  |  | 2 |  |  |  |  | 2 |
| 25 |  |  |  |  |  |  |  |  | - |
| 30 |  |  |  |  | 1 | 1 |  |  | 2 |
| 40 |  |  |  |  |  |  |  | **1** | 1 |
| Total (n) | 2 | 4 | 3 | 7 | 2 | 1 | - | 1 | 20 |
| n: Number of cases  Cases marked in **bold** indicate agreement | | | | | | | | | |

| **Table S2** Intraobserver variability for pathologist B - Whole tumour slide mean values of Ki-67 LI | | | | | | | | |
| --- | --- | --- | --- | --- | --- | --- | --- | --- |
| % | 0 | 5 | 10 | 15 | 20 | 25 | 30 | Total (n) |
| 0 | **2** | 4 |  |  |  |  |  | 6 |
| 5 |  | **2** | 1 | 1 |  |  |  | 4 |
| 10 |  | 1 | **2** | 1 |  |  |  | 4 |
| 15 |  |  | 1 | **1** | 1 |  |  | 3 |
| 20 |  |  |  |  | **1** |  |  | 1 |
| 25 |  |  |  |  |  |  | 1 | 1 |
| 30 |  |  |  |  |  | 1 |  | 1 |
| Total (n) | 2 | 7 | 4 | 3 | 2 | 1 | 1 | 20 |
| n: Number of cases  Cases marked in **bold** indicate agreement | | | | | | | | |

| **Table S3** Intraobserver variability for pathologist A – Hot spot values of Ki-67 LI | | | | | | | | | | | | |
| --- | --- | --- | --- | --- | --- | --- | --- | --- | --- | --- | --- | --- |
| % | 0 | 5 | 10 | 15 | 20 | 25 | 30 | 40 | 50 | 60 | 70 | Total (n) |
| 0 | **1** | 1 |  |  |  |  |  |  |  |  |  | 2 |
| 5 |  |  | 1 |  |  |  |  |  |  |  |  | 1 |
| 10 |  |  | **1** | 1 |  |  | 1 |  |  |  |  | 3 |
| 15 |  |  |  |  |  |  |  |  |  |  |  | - |
| 20 |  |  |  |  | **1** |  |  |  |  |  |  | 1 |
| 25 |  |  |  |  |  |  | 1 |  |  |  |  | 1 |
| 30 |  |  |  |  |  | 1 | **2** | 2 |  |  | 1 | 6 |
| 40 |  |  |  |  |  |  |  | **1** | 1 |  |  | 2 |
| 50 |  |  |  |  |  |  |  |  | **2** | 1 |  | 3 |
| 60 |  |  |  |  |  |  |  |  |  |  |  | - |
| 70 |  |  |  |  |  |  |  |  |  |  | **1** | 1 |
| Total (n) | 1 | 1 | 2 | 1 | 1 | 1 | 4 | 3 | 3 | 1 | 2 | 20 |
| n: Number of cases  Cases marked in **bold** indicate agreement | | | | | | | | | | | | |

| **Table S4** Intraobserver variability for pathologist B – Hot spot values of Ki-67 LI | | | | | | | | | | | | | |
| --- | --- | --- | --- | --- | --- | --- | --- | --- | --- | --- | --- | --- | --- |
| % | 0 | 5 | 10 | 15 | 20 | 25 | 30 | 40 | 50 | 60 | 70 | 80 | Total (n) |
| 0 | **2** | 1 |  |  |  |  |  |  |  |  |  |  | 3 |
| 5 |  | **2** |  |  |  | 1 |  |  |  |  |  |  | 3 |
| 10 |  |  |  | 1 |  | 1 |  |  |  |  |  |  | 2 |
| 15 |  |  |  | **1** |  |  |  |  |  |  |  |  | 1 |
| 20 |  |  |  | 1 |  |  | 1 |  |  |  |  |  | 2 |
| 25 |  |  |  |  | 1 |  |  |  |  |  |  | 1 | 2 |
| 30 |  |  |  |  | 2 |  |  |  | 1 |  |  |  | 3 |
| 40 |  |  |  |  | 1 |  |  | **1** |  |  |  |  | 2 |
| 50 |  |  |  |  |  |  |  |  | **1** |  |  |  | 1 |
| 60 |  |  |  |  |  |  |  |  | 1 |  |  |  | 1 |
| 70 |  |  |  |  |  |  |  |  |  |  |  |  | - |
| 80 |  |  |  |  |  |  |  |  |  |  |  |  | - |
| Total (n) | 2 | 3 | - | 3 | 4 | 2 | 1 | 1 | 3 | - | - | 1 | 20 |
| n: Number of cases  Cases marked in **bold** indicate agreement | | | | | | | | | | | | | |

| **Table S5** Intraobserver variability for the digital quantification of the Ki-67 LI (%) | | | | |
| --- | --- | --- | --- | --- |
|  | Whole tumour slide mean values | | Hot spot values | |
| Case no | 1^st^ quantification | 2^nd^ quantification | 1^st^ quantification | 2^nd^ quantification |
| 1 | 0.35 | 0.17 | 0.90 | 0.35 |
| 2 | 1.02 | 0.73 | 2.52 | 2.38 |
| 3 | 3.33 | 2.81 | 6.35 | 7.04 |
| 4 | 3.74 | 2.58 | 7.45 | 7.84 |
| 5 | 5.52 | 5.66 | 9.39 | 9.38 |
| 6 | 2.36 | 4.03 | 5.74 | 10.11 |
| 7 | 19.93 | 11.36 | 40.89 | 20.13 |
| 8 | 12.30 | 16.16 | 20.33 | 41.53 |
| 9 | 12.71 | 11.77 | 18.88 | 18.30 |
| 10 | 11.87 | 10.38 | 22.33 | 15.34 |
| 11 | 11.29 | 14.20 | 25.27 | 29.83 |
| 12 | 20.71 | 16.47 | 35.62 | 24.29 |
| 13 | 11.94 | 12.53 | 34.97 | 28.33 |
| 14 | 14.36 | 12.17 | 31.40 | 24.82 |
| 15 | 27.58 | 30.78 | 55.86 | 55.51 |
| 16 | 39.11 | 26.90 | 74.01 | 39.40 |
| 17 | 26.98 | 27.18 | 52.70 | 49.72 |
| 18 | 44.79 | 43.46 | 55.72 | 50.37 |
| 19 | 36.77 | 38.56 | 66.21 | 65.76 |
| 20 | 9.88 | 10.43 | 36.03 | 32.01 |
